# A case report of a pregnant woman infected with coronavirus disease 2019 pneumonia

**DOI:** 10.1097/MD.0000000000021335

**Published:** 2020-07-24

**Authors:** Jing Peng, Ruobing Li, Heng Yin, Fei Tang, Hui Xie, Min Li, Yun Zhao

**Affiliations:** a Department of Obstetrics, Maternal and Child Health Hospital of Hubei Province, Tongji Medical College, Huazhong University of Science and Technology; b Department of Gynecology and Obstetrics, Wuhan University of Science and Technology; c Department of Radiology, Maternal and Child Health Hospital of Hubei Province, Tongji Medical College, Huazhong University of Science and Technology, Hongshan District, Wuhan, China.

**Keywords:** coronavirus disease 2019, computed tomography, immunoglobulin G, immunoglobulin M, nucleic acid amplification testing

## Abstract

**Rationale:**

Since the end of December 2019, the outbreak of coronavirus disease 2019 (COVID-19) epidemic has occurred and spread rapidly throughout China. At present, China's epidemic situation has been basically controlled, but the number of cases worldwide is increasing day by day. On March 11, the WHO officially announced that the COVID-19 had become a global pandemic. However, there are currently limited data on pregnant women with COVID-19 pneumonia and their infants. In this paper, a case of a pregnant woman infected with COVID-19 pneumonia is reported.

**Patient concerns:**

We report a clinically confirmed COVID-19 pregnant woman. The patient was tested negative 4 times in nucleic acid test, but immunoglobulin G was positive and immunoglobulin M was negative before delivery, suggesting a previous infection.

**Diagnoses:**

The pregnant woman underwent a computed tomography scan of both lungs at 29 + 2 weeks of pregnancy, and scattered stiffness and frosted glass shadows of both lungs were observed. According to the diagnostic criteria for COVID-19 pneumonia in the “New Coronavirus Prevention and Control Plan Fifth Edition” of the National Health Commission of China, she was diagnosed as a clinically confirmed case.

**Interventions:**

The pregnant women received nebulized inhalation and oral cephalosporin treatment in a community hospital and was discharged after the symptoms disappeared. After that, she was isolated at home.

**Outcomes:**

The pregnant woman gave birth to a healthy baby after being cured from COVID-19 infection. The nucleic acid test of the neonatal pharyngeal swab was negative, and the neonatal serum test showed positive for immunoglobulin G and negative for immunoglobulin M.

**Lessons subsections:**

The findings of this case report are useful for understanding the possible clinical features of COVID-19 infection in pregnant women, the duration of the antibody, and passive immunity of the fetus.

## 1 Introduction

As of April 11, 2020, there have been more than 80,000 confirmed coronavirus disease 2019 (COVID-19) cases in China, including 3339 deaths.^[1]^ Through implementing a series of preventive control and medical treatment measures, the upward trend of the epidemic situation in China has been reversed, but the number of global infected cases is still growing.^[2]^ According to the World Health Organization’ s report on April 10, 2020, there have been more than 100 countries in the world suffering the outbreaks of COVID-19, and the number of confirmed cases worldwide has exceeded 1.5 million, including more than 90,000 deaths.^[3]^

During the epidemic, there is another special group that needs to be taken more care of, that is, pregnant women. The WHO-China Joint Mission Report introduced an investigation of 147 pregnant women cases (64 confirmed cases, 82 suspected cases, and 1 asymptomatic case) in China from February 16 to February 24, among which 8% were severe cases and 1% were critical cases.^[4]^ The mortality rate of the severe acute respiratory syndrome (SARS) and middle east respiratory syndrome epidemic was 15% and 27%, respectively. Pregnant women seem more likely to have a mild symptom of COVID-19 infection or to be an asymptomatic case, and the risk of COVID-19 infection for pregnant women may be much lower than SARS or middle east respiratory syndrome.^[5]^ The previous studies also show that pregnant women may be particularly vulnerable to COVID-19 infection.^[6]^ Thus, prevention and control of COVID-19 infection among pregnant women have become a major concern.^[7]^ In this paper, we will introduce a case report of a pregnant woman infected with COVID-19 pneumonia.

## 2 Case report

**Figure 1:**
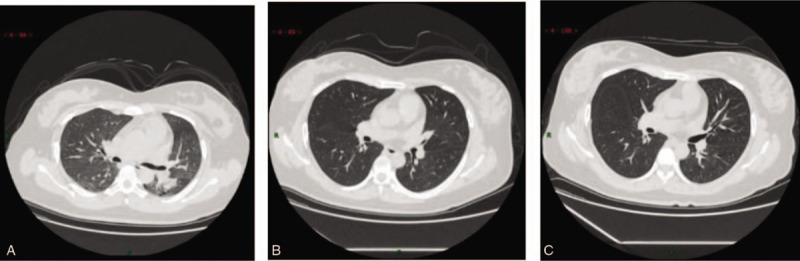
Chest CT scans of the pregnant woman: (A). January 30, 2020 CT: scattered consolidation of both lungs and ground glass shadow; (B). Mar 24, 2020 CT: most of the lung lesions are absorbed; (C). April 5, 2020 CT: further absorption of the lung lesions. CT = computed tomography.

A 33-year-old pregnant woman, whose last menstrual period was July 9, 2019, experienced her early pregnancy smoothly. Before January 23, 2020, she was doing fine and was in normal condition. Her colleague's father was hospitalized due to confirmed coronavirus pneumonia at the end of December 2019, and the colleague developed fever symptoms after taking care of his father in the hospital and was later confirmed with the sever acute respiratory syndrome coronavirus 2 (SARS-CoV-2) by quantitative real time polymerase chain reaction from throat swab. The pregnant woman with 28 + 5 weeks gestation developed cough and expectoration on January 26, 2020, and then had a fever on January 27 with her body temperature fluctuating between 37.5^°^C and 37.8^°^C. She was diagnosed with common pneumonia and treated by oral cephalosporin at a community hospital for 3 days. Her pulmonary computed tomography (CT) scan on January 30, 2020 (29 + 2 weeks gestation) (Fig. 1) showed scattered consolidation and ground-glass shadow of both lungs and the blood routine test results showed that her white blood cell count was 8.15 × 10^9^/L, neutrophil ratio was 78.6%, lymphocyte count was 1.08 ×10^9^/L, lymphocyte ratio was 13.3%, C-reactive protein was 99.67 mg/L, and hemoglobin was 115 g/L. At the same time, she was tested negative for influenza A and B virus antigens, Mycoplasma pneumoniae, and Chlamydia pneumoniae. Based on the criteria of COVID-19 pneumonia in the New Coronavirus Prevention and Control Program (5th edition) released by the National Health Commission of China on February 3, 2020,^[8]^ she was diagnosed as a clinically confirmed case, so quarantine treatment combined with nebulized inhalation and oral cephalosporin was applied for her. Five days later (30 weeks gestation), her body temperature returned to normal and the symptoms such as cough and expectoration disappeared. Subsequently, the pharyngeal swab test of SARS-CoV-2 revealed twice negative at a community hospital on March 5 and March 7, 2020 (34 + 4 weeks gestation). The pulmonary CT scan on March 24, 2020 (37 weeks gestation) (Fig. 1) revealed that most of the lung lesions were absorbed. On April 5, 2020 (38 + 5 weeks gestation), the pulmonary CT scan revealed further absorption of the lung lesions (Fig. 1), and the colloidal gold method revealed positive for immunoglobulin G (IgG) and negative for immunoglobulin M (IgM) in her serum. Moreover, 2 COVID-19 pharyngeal swab tests conducted on April 6, 2020 (the first day after delivery) and April 7, 2020 (the second day after delivery) showed negative results.

On April 5, 2020, at the gestational age of 38 weeks and 5 days, the pregnant woman was admitted to the hospital with small amount of vaginal bleeding. The admission check showed that her body temperature was 36.4^°^C, heart rate was 78 bpm, respiratory rate was 20 bpm, blood pressure was 111/61 mm Hg. No abnormalities were heard on cardiopulmonary auscultation. On the day of admission, she underwent cesarean delivery (CD) due to her previous CD in 2015. The operation went smoothly. The birth weight of the newborn was 3200 g, and the 1-minute and 5-minute Apgar scores were all 10. The newborn was tested with the COVID-19 Colloidal Gold method on the birth day by serum, and the test result was positive for IgG and negative for IgM. On April 7, 2020, the pharyngeal swab test for the newborn revealed negative. During hospitalization, she developed no fever, cough or other symptoms. Five days after CD, she was discharged.

The study was reviewed and approved by the Ethics Committee of Maternal and Child Health Hospital of Hubei Province, Tongji Medical College, Huazhong University of Science and Technology (Record number [2020] IEC[XM002]). All included women signed informed consent for therapeutic procedures and also for the publication of this case report.

## 3 Discussion

On March 11, 2020, WHO officially announced that the COVID-19 infection had become a global pandemic.^[9]^ Although CT and clinical manifestations and treatment monitoring of COVID-19 pneumonia patients have been extensively studied, there are few researches on pregnant women with COVID-19 pneumonia.^[10]^ It is highly important to systematically investigate the clinical characteristics and outcomes of pregnant women with COVID-19 to provide strong guidelines for prevention, treatment, and management.^[5]^

This case report investigates a pregnant woman who developed symptoms as early as January 26 and had typical signs of lungs on CT examination on January 30, 2020. She had a close contact with a confirmed COVID-19 pneumonia case within 14 days of onset, but did not accept throat swab nucleic acid test for SARS-CoV-2 at first. She was classified as a clinically diagnosed case at that time in Wuhan, China.^[8]^ Between March 5 and April 7, her pharyngeal swab test revealed negative for nucleic acid 4 times, and 3 chest CT scan images showed that the lung lesions were absorbed well. Moreover, she and her baby were tested positive for IgG and negative for IgM during hospitalization.

Considering the difference of sample site, the difference in experience of the operator, and the actual quantity of virus, there could be false negatives on occasion for oropharyngeal or nasopharyngeal swabs tests.^[11]^ Pulmonary CT scan plays an important role in diagnosis of COVID-19 infection and observation of therapeutic effect, and the typical imaging features is typically helpful in early screening of highly suspected cases.^[12]^ At first, the patient was misdiagnosed as common pneumonia without treatment in an isolation suite. If she continuously stays with other common patients, severe cross infection would happen among normal patients and medical staffs. During the outbreak of COVID-19, the diagnostic criteria have been adjusted to a certain extent, that is, no positive result of nucleic acid is required for clinical diagnosis of COVID-19 cases according to the fifth edition.^[8]^ The management of clinical confirmed cases was as same as laboratory confirmed cases, including quarantine at isolation suite of hospital for 2 weeks plus isolated at home for 2 weeks after cured from COVID-19 infection. For this case, it took 70 days from the onset of typical symptoms (fever and cough) to laboratory confirmation (IgG positive).

In a study involving 82 confirmed COVID-19cases and 58 suspected COVID-19 cases from China, the median duration of IgM testing was 5 days after the onset of symptoms (quartile range 3–6 days), and the median of IgG test was 14 days after the onset of symptoms (quartiles 10–18 days).^[13]^ Since the outbreak and spread of COVID-19 is rapid, there is no data on long-term immune response. Data on SARS-CoV-1 indicates that the titers of IgG and neutralizing antibodies peak 4 months after infection, and then decline at least 3 years after infection.^[13]^ The pregnant women and her baby were tested positive for IgG and negative for IgM, with a duration of 70 days from the onset to delivery.

After the first exposure to the virus, the patients with better immunity exhibit stronger body reactions. Innate immune cells play a vital role in effectively and proactively responding to various pathogens.^[14]^ Studies have shown that the maternal immune system is fully prepared to resist the invasion of foreign pathogens. For example, innate immune cells (such as NK cells and monocytes) respond more strongly to viral attacks, although some adaptive immune responses decrease during pregnancy, such as the decreased number of T and B cells.^[15]^ At the same time, under normal circumstances, IgG antibodies in maternal blood can be passed to the fetus through the placental barrier, allowing fetus to obtain passive immunity.^[16]^ Therefore, the positive result of fetal IgG may be due to the vertical transmission from the mother. In a study focusing on infants delivered by mothers with SARS, the lack of vertical transmission is considered to be the result of the development of passive immunity in late pregnancy, as evidenced by the presence of SARS-CoV-1 antibodies in the cord blood and breast milk of some pregnant woman.^[17]^

Considering the importance of this ongoing global public health emergency, although our conclusion is limited by the sample size, we believe that the findings of this case report are conducive for understanding the possible clinical features of COVID-19 infection in pregnant women, the duration of the antibody, and passive immunity of the fetus.

## Acknowledgments

The authors thank the obstetricians and radiologists for the diagnosis and treatment of pregnant women.

## Author contributions

**Conceptualization:** Min Li, Yun Zhao.

**Data curation:** Jing Peng, Ruobing Li, Heng Yin, Fei Tang, Hui Xie, Min Li, Yun Zhao.

**Visualization:** Hui Xie, Min Li.

**Writing – original draft:** Jing Peng, Ruobing Li, Min Li, Yun Zhao.

**Writing – review & editing:** Min Li, Yun Zhao.
